# Liver senescence in focus: Heterogeneity across aging and cancer

**DOI:** 10.1016/j.xgen.2026.101168

**Published:** 2026-02-11

**Authors:** Cleo L. Bishop

**Affiliations:** 1 Blizard Institute, Faculty of Medicine and Dentistry, Queen Mary University of London, 4 Newark Street, London E1 2AT, UK

## Abstract

How does senescent cell heterogeneity vary across different cell types in the liver in aging, fibrosis, and cancer? In *Cell Genomics*, Karpova and Li et al. reveal cell-type- and context-specific senescent cell signatures, offering the community a valuable resource and providing the potential for future therapeutic innovation.

## Main text

Cellular senescence is triggered by an array of cues and is underpinned by cells stably exiting the cell cycle. The key effectors of this fundamental cellular program are cyclin-dependent kinase inhibitors including p16^INK4A^ (p16) and/or p21^WAF1/CIP1^ (p21).^1^ The mechanistic consequences of senescence include altered morphological and cellular programs, which lead to an altered secretory phenotype, termed the senescence-associated secretory phenotype (SASP).^2^^,^^3^ In acute settings, senescent cells and their SASP have the potential to be beneficial; e.g., by promoting repair.^4^ However, with age, the accumulation and persistence of senescent cells can drive inflammation and tissue function, contributing to declining tissue function and chronic disease.^4^ It is now appreciated that senescent cell phenotypes are cell type and context dependent. As a consequence, senescence encompasses a spectrum of heterogeneity. Thus, understanding this cellular heterogeneity in a context-dependent manner is a key challenge in the senescence field.

**Figure 1:**
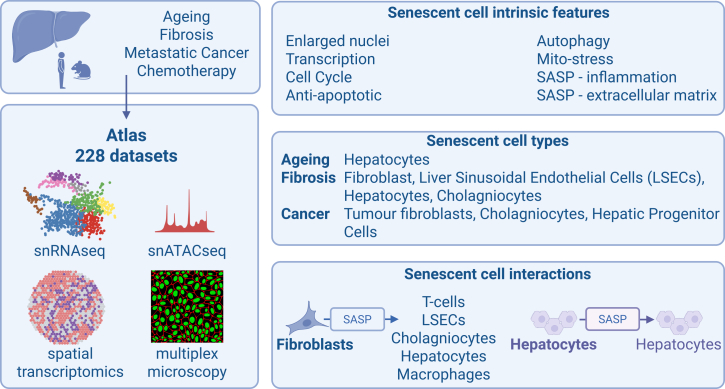
Schematic illustrating the key datasets and senescent cell intrinsic features, cell types, and cell-to-cell interactions in the liver in aging and disease Figure created in BioRender.

To carefully explore senescent heterogeneity within the liver, Karpova and Li et al.^5^ have leveraged a breadth of single cell and spatial technologies to unbiasedly profile senescent cell types in aging, chronic disease, and cancer (Figure 1). First, using snRNA-seq for young and old human livers (without cancer), the authors identified clusters of putative senescent (PS) hepatocytes; these clusters exhibited elevated expression of *CDKN1A* (encoding for p21), or *CDKN1A SERPINE1*. The prevalence of different clusters increased with donor age, fibrosis, or fibrotic liver disease.

The snRNA-seq data were then integrated with spatial transcriptomics and multiplex microscopy. This revealed that PS hepatocytes have larger nuclei as demonstrated for hepatic steatosis.^6^ Interestingly, the nuclei of PS hepatocytes expressing CDKN1A SERPINE1 were larger—irrespective of donor age—and were spatially clustered together within the tissue, suggesting that PS cells may form multicellular communities.

PS fibroblasts with *CDKN1A CCL2* expression were also identified with elevated abundance in donors with liver fibrosis and enlarged nuclei. Trajectory analysis suggests that PS fibroblasts could arise from portal fibroblasts or quiescent and activating hepatic stellate cells (HSCs). The authors also detect distinct PS cholangiocytes and liver sinusoidal endothelial cells (LSECs), each exhibiting unique molecular signatures and association with old age and fibrosis. The use of a small-bowel resection (SBR) mouse model revealed similar signatures within PS hepatocyte, endothelial, fibroblast, and cholangiocyte.

Spatial transcriptomic data identified five sub-niches of multicellular communities of PS hepatocytes. These niches had specific associations with fibrosis, indicating distinct spatial remodeling of portal versus parenchymal compartments during fibrosis progression. Exploration of two older fibrotic samples identified PS fibroblasts as signaling hubs influencing extracellular remodeling, and CXCL12-CXCR4 mediated crosstalk with T cells and inflammatory macrophages within the senescent microenvironment. These observations were recapitulated in the SBR model, indicating a conserved role for PS fibroblast communication with the immune compartment during fibrosis. More broadly, these findings raise the possibility of collaborative intercellular crosstalk between senescent cells and their neighbors that initiates, maintains, and amplifies inflammation and extracellular remodeling within the fibrotic tissue microenvironment.

When normal liver was compared with metastatic colorectal cancer (mCRC) in the liver, similar clusters of PS cells across a range of cell types were detected. However, in mCRC, some of these groups were more abundant (e.g., PS hepatocytes CDKN1A, PS hepatocytes CDKN1A SERPINE1, and a subset of the PS LSECs) and displayed altered transcriptional profiles relative to normal tissue. Other groups (e.g., PS fibroblasts and cholangiocytes) were comparable both in abundance and gene expression signatures. Unique mCRC signatures included activated HSCs, which shared gene signatures with PS fibroblasts, and hepatic progenitor cells (HPCs) and cholangiocytes expressing *CDKN2A/CDKN2B*, with unique SASP factors detected in the CDKN2A+ (p16) clusters. Intriguingly, both metastatic cancer and, to a greater extent, chemotherapy increased senescent cell burden. Beyond the tumor, two PS cancer-associated fibroblast (CAF) populations were observed: those expressing CDKN2A/2B and those expressing CDKN1A SERPINE1; the latter secreted more SASP factors with trajectory analysis suggesting that CAFs located within the tumor may derive from PS portal fibroblasts located outside the tumor.

Finally, the authors integrated their data with clinical features, identifying PS groups associated with aging (PS hepatocytes CDKN1A SERPINE1 and PS LSECs CDKN1A) and those associated with fibrosis (all other PS cell types). They define shared molecular programs, implicating the SASP regulator *NFKB1* and p21 regulators *KLF6* and *ATF3* together with stress and survival-associated genes. The datasets were also used to produce a liver SASP. PS fibroblasts and LSECs shared common features, while PS epithelial cells exhibited a distinct SASP signature. Interestingly, samples with a high burden of senescent cells had high levels of intercellular crosstalk with PS fibroblasts highlighted as having the most outward and inward intercellular communication. Both human and mouse PS fibroblasts had thrombospondin signaling as a the top enriched pathway, demonstrating cross-species conservation.

Taken together, Karpova and Li et al. utilized cutting-edge technologies at the single cell, transcriptional, protein, and spatial levels to systematically and unbiasedly explore senescent cell signatures in the liver as a consequence of age, fibrosis, metastatic cancer, and chemotherapy treatment. This work provides deep insights into senescent cell signatures within and between different cell types across a breadth of contexts. Spatially, morphological changes, in particular enlarged nuclei, are a common senescent cell feature observed across cell types. Within the liver, they find that CDKN1A is a key senescent biomarker and reveal that senescent fibroblasts have a high level of bidirectional communication with their neighbors. Beyond these fundamental insights, the authors provide a treasure trove of multi-omics and spatial data of great interest and value to the community, inspiring curiosity-driven questions and driving therapeutic innovation.

## Acknowledgments

C.B. is grateful for funding from QMUL Impact Fund, Barts Charity (G-003062), Biotechnology and Biological Sciences Research Council (UKRI3174), Medical Research Council (UKRI583), Unilever, ValiRx, and Wellcome Trust (223803/Z/21/Z).

## Declaration of interests

C.B. has acted as a consultant for Senisca, StarkAge, and AKLRD. C.B.’s laboratory received funding from ValiRx.
